# Overwhelmed by beauty and faith: review on artistic and religious travelers’ syndromes

**DOI:** 10.3389/fnbeh.2024.1341845

**Published:** 2024-02-27

**Authors:** Damaris Cisneros-Ceh, Darina Esquivel-Heredia, Allan Medina-Vargas, Hugo Azcorra-Perez, Claudia Guadalupe Chi-Mendez, Alina D. Marin-Cardenas, Nina Mendez-Dominguez

**Affiliations:** ^1^School of Medicine, Faculty of Medicine, Autonomous University of Yucatan, Merida, Yucatan, Mexico; ^2^Centro de Investigaciones Silvio Zavala, Universidad Modelo, Merida, Yucatan, Mexico; ^3^Hospital Regional de Alta Especialidad de la Península de Yucatan, IMSS-Bienestar, Merida, Yucatan, Mexico

**Keywords:** Stendhal syndrome, Jerusalem syndrome, travelers’ syndromes, ecstatic epilepsy, psychoemotional ecstasy

## Abstract

Traveling with the intention of encountering art or seeking purification of the spirit involves retribution of intangible nature and therefore can be expected to be a positive experience; nevertheless, among susceptible travelers, there is also a possibility of experiencing pathological conditions. Although it is colloquially known that beauty lies in the eyes of the beholder, it is necessary to mention that the appreciation of beauty, immensity, or mysticism contained in masterpieces is not perceived only through the eyes but through other sense organs as well. Additionally, this is understood within a cultural framework and through previous knowledge. The reaction triggers a series of somatosensory responses of diverse nature, with a wide range of responses that together constitute a pathological phenomenon that can be defined as syndromic by eliciting signs and symptoms of a physical, physiological, and psychotic nature. Both Stendhal and Jerusalem syndromes are travelers’ syndromes that may occur in response to objectively aesthetic elements saturated with meaning linked to the cultural heritage of contemporary humanity. While Stendhal syndrome evokes physical and psychoemotional symptoms from the contemplation of art, Jerusalem syndrome goes beyond perception, adding delusions of being a religious or prophetic protagonist pursuing individual or collective salvation.

## 1 Introduction

Travelers’ syndromes include Stendhal syndrome and Jerusalem syndrome, which manifest as psychological symptoms, perceptual disorders, persecutory feelings, states of anxiety, depressive feelings, or euphoria. Sometimes, there may be an omnipotent thought and an absence of self-criticism where the patient usually feels disoriented and confused (Beyle, 1999; Bauer, 2021).

### 1.1 Travelers’ syndromes

Travelers’ syndromes are multisystemic pathological responses elicited during a trip or while visiting foreign places. Far from truly corresponding to a medical or psychiatric condition, these syndromes can be understood as a phenomenon of either subjective or objective nature. Distinctions should be made when classifying the experiences of summer beachside tourists and those of cultural, religious, and artistic travelers, as the intentions for their voyages are different.

Sun, sand, and beach-going tourists may develop “Hawaii syndrome,” which is characterized by anxiety when trying to untangle profound aspects of historic importance in an otherwise relaxing vacation (Thomas et al., 2005). Contrastingly, beachside resort visitors may fall into the “coconuts and bananas syndrome” characterized by adopting an overly uncomplicated routine in colonial, coastal, or island destinations, as explained by Streltzer, even when similar conditions can also be found under the name of Caribbean or island syndromes (Tamura et al., 2001; Thomas et al., 2005; Dusse et al., 2017). Tourists may hold prejudices against native people and intrinsically qualify them as lazy, or deceptive, leading them to adopt a distrusting attitude that adds stress and discomfort during their vacation. In highly popular beaches, “crowding syndrome” can happen when seasonal occupancy exceeds capacity, and when traveling on a limited budget, vacationers can experience “economic class syndrome,” which involves pain and fatigue from an unpleasant trip (Tamura et al., 2001; Thomas et al., 2005).

However, artistic and spiritual voyagers differ in purpose from beach-going tourists as they are planned by an individual or a group with the shared intention to encounter a destination saturated with cultural, symbolic, or mystical significance. The appreciation of such destinations begins long before the journey itself because visitors hold a tacit knowledge that endows them to conceive the meaning of the elements to be encountered at their destination (Palacios-Sánchez et al., 2018). In the present review, the authors explore the pathological implications related to artistic and spiritual travels.

### 1.2 Artistic and religious travelers’ syndromes

The significance and symbolism of artistic and religious places can be partially understood through the term *´ilm*, which, as explained by Akkach (2019), involves a tacit perception of the artistic and religious meaning of different elements of Islamic places (Akkach, 2019). While this perception of aesthetics can lead to joy, it can also produce sadness, satisfaction, or even euphoria. Artistic and religious travelers find, within art pieces, architecture, and landscapes, an intersection between their feelings and the objective and subjective traits present in emblematic places.

There are several artistic travelers’ syndromes in modern literature named after the places that trigger such psychoemotional responses in visitors due to their unique beauty, architecture, and overall ambience. These include, but are not limited to, Florence syndrome, Saint Petersburgh syndrome, Paris syndrome, and Tokyo syndrome, among others. Instead of aiming to describe the beauty and cultural values of exemplary cities, the intention of the present review is to explore the intrinsic susceptibilities and manifestations in artistic and religious travelers experiencing these syndromes, and for this purpose, we focused on Stendhal and Jerusalem syndromes as they broadly represent artistic and religious travelers, respectively. While Stendhal syndrome occurs in a merely idle individual, Jerusalem syndrome can modify the affected person’s behavior (Arias, 2019; Brielmann et al., 2021; Marinho et al., 2021; Firouzbakht et al., 2022).

### 1.3 Stendhal syndrome

The history and name of the Stendhal syndrome are derived from the famous 19th century French novelist Marie Henri Beyle, whose pseudonym was Stendhal (Beyle, 1999). However, the first person to use the term Stendhal syndrome was psychiatrist Graziella Maghereni. According to Airault (2015), there are two facilitators of the syndrome which include being before the original work, the connotation that the art piece or the author as perceived by the affected individual, and a lack of attenuating elements.

There is evidence of three syndromic types: (1) predominant disorders of thought, (2) predominant disorders of affect, and (3) panic attacks or somatic projections of distress. Each of these categories has different reactions in exposed individuals. The first type is related to alterations in the perception of colors and sounds, which cause feelings of guilt and anxiety. The second type generates anguish; feelings of inferiority, precariousness, or insufficiency; superiority; euphoria; and exaltation, in addition to a loss of the criterion of reality (Beyle, 1999). Finally, the third type is related to panic or fainting attacks, tachycardia, epigastric discomfort, and chest pain (Bauer, 2021). For the three syndromic types, repressed sexual drives, fatigue, insufficient sleep, the end of a trip, and vital moments of uncertainty or change are identified as potentially triggering factors (Bauer, 2021).

In general, Stendhal syndrome can be explained as a state of mind that manifests when works of art of remarkable beauty and radiance are observed. The environment in which an individual is raised is relevant to their mental condition since the physical environment can trigger or mitigate mental disorders. This explains why more cases of this syndrome have been observed in Florence, a tourist city renowned for its great beauty (Firouzbakht et al., 2022). Regarding treatment, the evidence shows that it does not go beyond the prevention of major lesions after syncopal activity and rules out associated major complications or comorbidities (Serrano, 2020).

Symbolism, magic, and religion have accompanied human beings in their development within the environment (Cutsforth-Gregory, 2020). The appearance of psychotic episodes influenced by mysticism and symbolism in religion began to be recorded when Stendhal narrated his feelings; however, the manifestations can be traced back to much earlier. Visitors who appreciate pieces of art or religious symbols of great significance may present the symptoms, turning the impact of perceived beauty into psychotic events of uncontrolled anxiety and other manifestations (Palacios-Sánchez et al., 2018).

The authors define patients with Stendhal syndrome as passively suffering from their minds and bodies’ reactions to art and beauty, with no necessary association with religion. However, in one case described by Mishima (2010), Stendhal syndrome triggered not only a cumulus of emotions but also the actions of a Buddhist monk, who, as explained by Lambert (2014), was so overwhelmed by the magnificence of The Temple of the Golden Pavilion that such feelings drove him to set the temple on fire in a need to experience the mourning of living in a time where such beauty no longer exists.

Even when related to a sacred place, the motivation behind the monk’s act was not mystic or religious, which means that his behavior was unrelated to the salvation of human souls and Jerusalem syndrome. It remains a mystery what turns an individual with Stendhal syndrome from passively afflicted to actively affected. However, in the case of the monk, it may relate to idiosyncrasy or intentions.

### 1.4 Jerusalem syndrome

Jerusalem syndrome was first described in 1937 by the psychiatrist Ezrath Nashim, who observed an increase in the number of people suffering from psychiatric decompensation when visiting Israel, describing it as the “Jerusalem fever.” (Picard et al., 2021) He asserted that the city has a unique attraction for religious or faith-attached people, and the people primarily affected are those affiliated with the Jewish and Christian faiths (Picard et al., 2021). Regarding its epidemiology, approximately during 1999, several cases of Jerusalem syndrome were most expected by medical personnel in Jerusalem and surrounding regions because, under Judaic and Christian traditions, the avengement of the millennia entails closeness to apocalyptic times, and consequently, such times invoke actions such as pilgrimage as a ritual to prepare religious people for the final judgment and entry into a holy afterlife. To date, the annual frequency of people affected was an average of 100 tourists, with nearly 40% of them requiring hospitalization (Yair et al., 2000).

Far from being restricted to psychoneurological manifestations, Jerusalem syndrome also relates to pilgrimage and fasting. Its name reflects the atypical conduct and the subjective response experienced by individuals when traveling to sacred, spiritual, or mystical places. The term can be extensive for defining others, such as the India syndrome and the Way of Saint James syndrome, because they share similar faiths and religious beliefs. Fasting may be interpreted by religious individuals as a purifying phase, akin to spiritual self-starvation (Latzer and Stein, 2016; Picard, 2023).

Underlying psychopathologies are common among persons who have experienced Jerusalem syndrome, therefore, the syndrome can be presented in three types: (1) with an underlying psychological disorder; (2) with overlapping delusional ideas; and (3) mild manifestations, uncomplicated by previous psychopathology. In all three types, the affected person has a religious background (Prochwicz and Sobczyk, 2011; Poleszczyk and Swiecicki, 2013; Butler, 2016; Latzer and Stein, 2016).

Type 1 can be identified in individuals who believe that they are living a quest that needs to be fulfilled before leaving the city. People who go on pilgrimages can attempt journeys to satisfy their religious duty or to renew and purify their souls. The perception of having a higher mission related to an immortal soul provides patients with an obsessive conduct but also a psychotic one. The patients may identify themselves as a biblical character and, therefore, start acting like him/her, prophesizing and citing passages from a sacred book.

In type 2, the affected persons may not have any known antecedent of mental illness, nevertheless, these persons hold strict, obsessive spiritual and religious ideas. These visitors with type 2 Jerusalem syndrome typically feel exalted when visiting the biblical places and persons from cults or small religious group with distinctive rites and beliefs may be more susceptible.

Patients with type 3 are individuals without psychological disorders who may experience a brief psychotic episode, which can be prevented or treated when symptoms are present.

In one case report, a patient presented after self-mutilation, assuring to be reborn and pure. In his discourse he explained that according to the biblical passages, only by being reborn men should enter heaven. The patient interpreted that in his genitals remained the cause for sin and thus, having genitals would prevent him from reaching heaven in afterlife (Zislin et al., 2002).

The natural history of the syndrome can be broken down into seven stages. The first stage is characterized by agitation, feelings of tension, and anxiety. In the second stage, an individual supernatural call is perceived, thus the affected person wants to leave his tour group or family and stroll around the city alone.

In the third stage, the purification obsession begins, and the visitor suffers from an urge to be clean and pure. Most commonly, the traveler may wash, bathe, cut their nails, and repeat cleansing rituals again and again. In one case report of a patient with Jerusalem syndrome, the patient self-mutilated to achieve purification and eliminate a source of sin. By the fourth stage, the patients search for more expressions of purity and tend to wear clean white robes.

In the fifth stage, the travelers cannot contain their commitment to pursue a mission any longer and start signing and shouting religious songs, verses, and psalms. To reach their objective, the patients embark on a procession to visit holy places, and by the seventh stage, they start preaching in those holy places, but their speech is usually incoherent (Wilson, 2019; Fusar-Poli et al., 2022; Şahin et al., 2022).

The symptomatology of this condition is similar to that of Stendhal syndrome at the initial stage, as individuals present with anxiety reaching the psychotic dimension, tachycardia, agitation, nervousness, tension, dizziness, and obsession. These symptoms are usually associated with previous psychological and psychiatric ailments that manifest as a result of visiting Israel (Li et al., 2022; Szuhany and Simon, 2022; Wickramasekera and Tubeuf, 2023). Based on our review, Stendhal syndrome is widely associated with this syndrome because it also generates emotional ecstasy and a disorder of the perception of reality, since individuals are not aware of their true identity during the presentation of psychotic symptoms. But it has also been suggested that in most cases, the travel may be the trigger but not the cause, as the affected persons may be already holding a delusionary system related to religion or arts for planning the trip in the first place (Guerrero et al., 2010; Exline and Wilt, 2023).

### 1.5 Connections between aesthetics, art, and religious perception

Perceivable traits and characteristics of artistic objects and emblematic places may generate a sense of connection of a larger implication that can be perceived by sensorimotor receptors; it is a contemporary way of bonding past and future and physical and spiritual components in a mindful way through aesthetic experiences or as contemplative states. Djebbara (2023) explained the bondage between neuroaesthetics and contemplative practices by propounding that, as a whole, aesthetic contemplative states translate into admiration and a sense of relatedness (Djebbara, 2023; Djebbara et al., 2024). Perhaps, at least in part, this perceived embedding in a greater sense can drive pathological manifestations, such as those of Jerusalem syndrome, when susceptible individuals adopt a sacred duty or when they attempt to wear robes, physically resembling characters in religious texts.

However, regarding susceptibility, it is important to mention that contemplative states can be either intentional or unintentional; however, in any case, susceptibility can come from pathological conditions. Non-pathological contemplative states can start from the observer (IN-I) or from the artistic element (EX-I), and the presence or the absence of a deliberate intention to center attention on such elements makes the difference; in EX-I, the element calls attention without intention, but in IN-I, the viewer intentionally focuses on the element. In planned visits, artistic travelers’ syndromes appear to come from IN-I (Charalambous and Djebbara, 2023).

**Figure 1:**
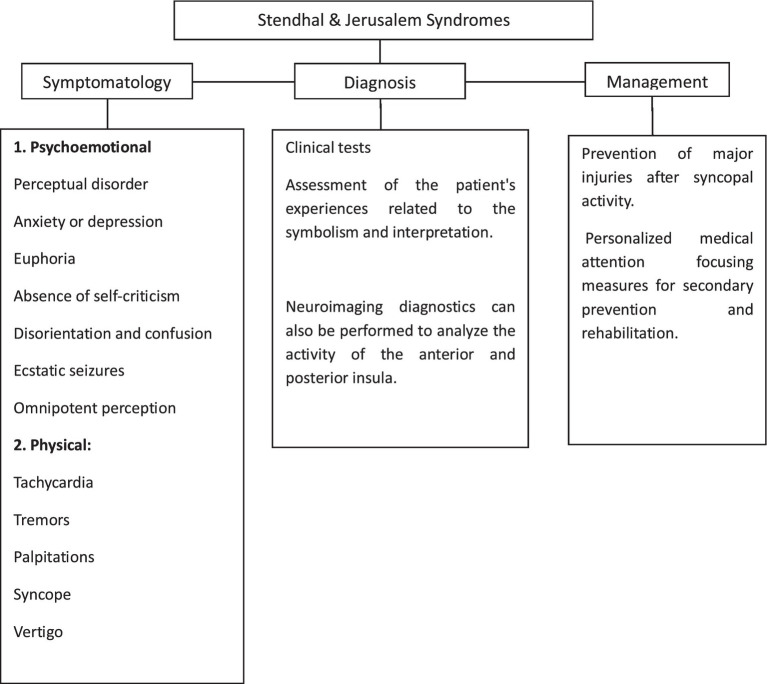
Characteristics of travelers’ syndromes.

Pathological susceptibility may relate to underlying disorders as identified in a survey on Stendhal syndrome, where the authors found that 17% of respondents confessed to having high degrees of melancholy and anxiety at the time of the trip (Butler, 2016); 66% had experienced predominant disorders of thought, including alterations in perception, persecutory feelings, and anxiety; additionally, 29% presented disorders of affections, with a variable degree of depressive behaviors or feelings of superiority; and another 5% experienced panic attacks or somatic projections of distress (Figure 1) (Guerrero et al., 2010). Jerusalem syndrome includes all these aspects but is more commonly linked to people with prior mental conditions and while recurrences have been reported in both Stendhal syndrome and Jerusalem syndrome, only a few cases of repetition can be found in the literature mainly because the key recommendation is always to withdraw the patient from the city that triggers the malaise (Buckley, 2023).

### 1.6 The digital age: preparation before traveling

According to 4E cognitive scientists, cognition exceeds the physical limits of the human brain because human minds extend to elements embedded in natural and cultural settings and expand with the structures that recall a meaning or a memory, and by recalling it, its connotation is amplified. Artworks are essentially filled with symbolism, where color combinations, shapes, and any represented persons pertain to a period; a time; or a social, religious, or historical moment, and for this reason, symbolism in art can enable human minds to extend their perception of symbolism to other elements and structures (Newen et al., 2018). Masterpieces encountered by tourists in emblematic places can be conceived as core structures of extracranial cognition. By familiarizing themselves with other travelers’ reviews, photos, and discourse on their experiences during their visit to emblematic cities or a specific artwork, contemporary tourists from the post-pandemic era can broaden their cognition in preparation for their trip.

Parting from the embedded, embodied, extended, and enactive approaches of the 4E cognition, McCauley (2023) proposed that emotional and evolutive approaches are also valid. As religious representations are related to ontological comprehension and are retained in the memory, they can be accessed, for example, by admiring symbolic art pieces or sacred structures during a trip. All related and accompanying emotional and evolutionary inferences from meaningful elements may automatically come together, contributing to a cumulus of sensations, which may even translate into symptoms among susceptible individuals, as explained in the artistic travelers’ syndrome description (McCauley, 2023). Aagaard (2021) criticized the assumption of an all-too-harmonic conceptualization of 4E cognition when referring to the human–technology interconnections, as it appears to be tamely accepted. Technology can serve as an extracorporeal repository of human knowledge; specific data contained in technological devices can orient a decision; but contrastingly, if data are accessed without comprehension, contextualization, or correct interpretation, the human–technology dyad lacks harmony and the benefit from this bonding is limited (Aagaard, 2021). However, in the case of Stendhal syndrome, as an undesirable manifestation, web-based content and technology may perhaps benefit tourists by offering not only idealistic, platonic descriptions but also negative reviews of common and diverse travelers.

The photographic and screen-based observation of emblematic masterpieces carried out by individuals can generate a cascade of emotions and prepare them for the encounter (Zislin et al., 2002; Aljunaidy and Adi, 2021). An individual’s affective affinity with a photograph or film of a landscape, painting, or any figment of the imagination is an emotional event for the observer (Djebbara, 2023). Social networks occupy a large part of contemporary societies; they are a way of socializing and learning about the world as they serve as a means of traveling without leaving home. Images, videos, and virtual reality have an impact on the nervous system, which mitigates the neurohormonal response of the hypothalamus to physical exposure to art, exotic landscapes, and religion, as digital resources generate a response. Therefore, after careful consideration of mental health history, in an individual approach to those planning to embark on an artistic or religious trip, it remains an option to previously expose the individual to digital and technological resources. Additionally, encouraging them to carry pertinent medication in case of an unwanted response during their visit may be beneficial. Individuals who have already suffered from these syndromes may be prevented from traveling knowing that if no measures are taken, they may fall ill again.

## 2 Conclusion

Under the classification of travelers’ syndromes, Stendhal and Jerusalem syndromes serve as prototypes for their respective classes because Stendhal syndrome pertains to the symptomatology experienced by artistic and religious travelers, while Jerusalem syndrome involves a staged cascade of emotions and actions guided by religious beliefs driven by the urge to purify and embark in a sacred mission.

Although Stendhal syndrome is not exclusive to Florence, Paris, or cities with rich exhibitions of art and culture, it can also occur to individuals visiting churches, temples, or buildings of laic exuberance as long as it remains unrelated to a religious predicament, which explains why even atheists may develop Stendhal syndrome in sacred places.

Stendhal syndrome often presents passively, while Jerusalem syndrome involves oriented behaviors and conduct. It is worth noting that susceptibility to Stendhal syndrome is linked to the coexistence of psychological conditions. Underlying psychiatric conditions are common in Jerusalem syndrome; in the urge to pursue a supernatural afterlife retribution, the need for purification can translate into a self-injury of varying severity. It is crucial for health personnel to identify the motivations for self-aggression as they are perceived as a necessary step for the greater good by the affected patients, and therefore, therapy should be directed toward addressing this cause. Finally, symptoms such as tachycardia, ecstatic seizures, dizziness, disorientation, alterations in perception and thinking, and confusion were found in both syndromes.

A proposed means of mitigating the overwhelming impression that visitors may experience at emblematic places is to utilize photographic and web-based means, including screen devices and virtual reality. This approach can help facilitate a controlled response and prevent a dramatic reaction while visiting the wonders of our precious world.

## Author contributions

DC-C: Conceptualization, Investigation, Writing – original draft. DE-H: Conceptualization, Investigation, Writing – original draft. AM-V: Conceptualization, Investigation, Writing – original draft. HA-P: Methodology, Supervision, Writing – review & editing, Visualization. CC-M: Investigation, Writing – review & editing. AM-C: _. NM-D: Resources, Supervision, Writing – review & editing.

## References

[ref1] Aagaard J. (2021). 4E cognition and the dogma of harmony. Philos. Psychol. 34, 165–181. doi: 10.1080/09515089.2020.1845640

[ref2] Airault R. (2015). Risks of psychiatric decompensation in travel. Rev. Prat. 65, 509–512. PMID: 26058196

[ref19] Akkach S. (2019). Ilm: Science, religion and art in Islam. Adelaide, South Australia: University of Adelaide Press. doi: 10.20851/ilm-1

[ref3] Aljunaidy M. M. Adi M. N. (2021). Architecture and mental disorders: a systematic study of peer-reviewed literature. HERD 14, 320–330. doi: 10.1177/1937586720973767 33356588

[ref4] Arias M. (2019). Neurology of ecstasy and surrounding phenomena: ecstatic, orgasmic and musicogenic epilepsy. Stendhal syndrome. Autoscopic phenomena. Neurology 34, 55–61. doi: 10.1016/J.NRL.2016.04.010, PMID: 27340019

[ref5] Bauer I. L. (2021). Death as attraction: the role of travel medicine and psychological travel health care in 'dark tourism'. Trop. Dis. Travel Med. Vaccines 7:24. doi: 10.1186/s40794-021-00149-z, PMID: 34380578 PMC8359045

[ref6] Beyle M. Rome, Naples, and Florence. 3rd Valencia: Pre-textos; (1999)

[ref7] Brielmann A. A. Nuzzo A. Pelli D. G. (2021). Beauty, the feeling. Acta Psychol. 219:103365. doi: 10.1016/j.actpsy.2021.103365, PMID: 34246875 PMC8514293

[ref8] Buckley R. (2023). Tourism and mental health: foundations, frameworks, and futures. J. Travel Res. 62, 3–20. doi: 10.1177/00472875221087669

[ref9] Butler B. (2016). The efficacies of heritage: syndromes, Magics, and Possessional acts. Public Archaeol. 15, 113–135. doi: 10.1080/14655187.2016.1398390

[ref10] Charalambous E. Djebbara Z. (2023). On natural attunement: shared rhythms between the brain and the environment. Neurosci. Biobehav. Rev. 155:105438. doi: 10.1016/j.neubiorev.2023.105438, PMID: 37898445

[ref11] Cutsforth-Gregory J. K. (2020). Postural tachycardia syndrome and Neurally mediated Syncope. Continuum 26, 93–115. doi: 10.1212/CON.0000000000000818 31996624

[ref12] Djebbara Z. (2023). “Rhythms of the brain, body and environment: a neuroscientific perspective on atmospheres” in Designing atmospheres: Theory and science. eds. Canepa E. Condia B. (Manhattan: New Prairie Press)

[ref13] Djebbara Z. King J. Ebadi A. Nakamura Y. Bermudez J. (2024). Contemplative neuroaesthetics and architecture: a sensorimotor exploration. Front. Archit. Res. 13, 97–111. doi: 10.1016/j.foar.2023.10.005

[ref14] Dusse L. M. S. Silva M. V. F. Freitas L. G. Marcolino M. S. Carvalho M. D. G. (2017). Economy class syndrome: what is it and who are the individuals at risk? Rev. Bras. Hematol. Hemoter. 39, 349–353. doi: 10.1016/j.bjhh.2017.05.001, PMID: 29150108 PMC5693389

[ref15] Exline J. J. Wilt J. A. (2023). Supernatural attributions: seeing god, the devil, demons, spirits, fate, and karma as causes of events. Annu. Rev. Clin. Psychol. 19, 461–487. doi: 10.1146/annurev-clinpsy-080921-081114, PMID: 36480930

[ref16] Firouzbakht T. Shen M. L. Groppelli A. Brignole M. Shen W. K. (2022). Step-by-step guide to creating the best syncope units: from combined United States and European experiences. Auton. Neurosci. 239:102950. doi: 10.1016/j.autneu.2022.102950, PMID: 35158162

[ref17] Fusar-Poli P. Salazar de Pablo G. Rajkumar R. P. López-Díaz Á. Malhotra S. Heckers S.. (2022). Diagnosis, prognosis, and treatment of brief psychotic episodes: a review and research agenda. Lancet. Psychiatry 9, 72–83. doi: 10.1016/S2215-0366(21)00121-8, PMID: 34856200

[ref18] Guerrero A. L. Barceló A. Ezpeleta D. (2010). Stendhal syndrome: origin, nature, and presentation in a group of neurologists. Neurology 25, 349–356. doi: 10.1016/J.NRL.2010.02.004, PMID: 20738954

[ref21] Lambert L. (2014). The Funambulist Pamphlets: Spinoza. Brooklin, New York: Punctum books.

[ref22] Latzer Y. Stein D. (Eds.) (2016). Bio-psycho-social contributions to understanding eating disorders. Switzerland: Springer International Publishing.

[ref23] Li W. Zhao Z. Chen D. Peng Y. Lu Z. (2022). Prevalence and associated factors of depression and anxiety symptoms among college students: a systematic review and meta-analysis. J. Child Psychol. Psychiatry 63, 1222–1230. doi: 10.1111/jcpp.13606, PMID: 35297041

[ref1001] Marinho G. Peta J. Pereira J. Marguilho M. (2021). Stendhal syndrome: Can art make you ill?. Europ. Psychi. 64:S317. doi: 10.1192/j.eurpsy.2021.852

[ref24] McCauley R. N. (2023). Darwinian bases of religious meaning: interactionism, general interpretive theories, and 6E cognitive science. J. Cogn. Cult. 23, 1–28. doi: 10.1163/15685373-12340149

[ref1002] Mishima Y. (2010). The Temple of the Golden Pavilion. London, UK: Random House Publishing.

[ref25] Newen A. Bruin L. De Gallagher S., editors. The Oxford handbook of 4E cognition. United States: Oxford University Press; (2018).

[ref26] Palacios-Sánchez L. Botero-Meneses J. S. Pachón R. P. Hernández L. B. P. Triana-Melo J. D. P. Ramírez-Rodríguez S. (2018). Stendhal syndrome: a clinical and historical overview. Arq. Neuropsiquiatr. 76, 120–123. doi: 10.1590/0004-282X20170189, PMID: 29489968

[ref27] Picard F. (2023). Ecstatic or mystical experience through epilepsy. J. Cogn. Neurosci. 35, 1372–1381. doi: 10.1162/jocn_a_02031, PMID: 37432752 PMC10513764

[ref28] Picard F. Bossaerts P. Bartolomei F. (2021). Epilepsy and ecstatic experiences: the role of the insula. Brain Sci. 11:1384. doi: 10.3390/brainsci11111384, PMID: 34827383 PMC8615543

[ref29] Poleszczyk A. Swiecicki Ł. (2013). Syndrom jerozolimski - opis przypadku [Jerusalem syndrome - a case report]. Psychiatr. Pol. 47, 353–360. PMID: 23888767

[ref30] Prochwicz K. Sobczyk A. (2011). Syndrom jerozolimski. Objawy, przebieg i kontekst kulturowy. Psychiatr. Pol. 45, 289–296. 21714216

[ref31] Şahin F. Candansayar S. Geniş B. (2022). Jerusalem syndrome: a case displaying similar symptoms to Jerusalem syndrome during Mecca visit. Turk Psikiyatri Derg. 33, 290–292. doi: 10.5080/u26966.G 36592108

[ref32] Serrano G. X. (2020). Domes, frescoes, sculptures, facades, landscapes, religious centers, and the saturation of artistic beauty can be triggers of the disturbing neurological condition called Stendhal syndrome or traveler's syndrome. Div Cient. 1, 2–5.

[ref33] Szuhany K. L. Simon N. M. (2022). Anxiety disorders: a review. JAMA 328, 2431–2445. doi: 10.1001/jama.2022.22744 36573969

[ref20] Tamura K. Kurabayashi H. Kubota K. (2001). Acute disease onset and economy class syndrome of the tourist in the hot spring resort. J. Japan Soc Climatol. Phys. Med. 64, 164–165. doi: 10.11390/onki1962.64.141

[ref34] Thomas R. N. Pigozzi B. W. Sambrook R. A. (2005). Tourist carrying capacity measures: crowding syndrome in the Caribbean. Prof. Geogr. 57, 13–20. doi: 10.1111/j.0033-0124.2005.00455.x

[ref35] Wickramasekera N. Tubeuf S. (2023). Measuring quality of life for people with common mental health problems. J. Ment. Health 32, 3–10. doi: 10.1080/09638237.2020.1818190 32915686

[ref36] Wilson C. (2019). Beyond Jerusalem syndrome: religious mania and miracle cures. Jerusalem Quart. 78, 16–37.

[ref37] Yair B.-E. Durst R. Katz G. Zislin J. Strauss Z. Knobler H. (2000). Jerusalem syndrome. Br. J. Psychiatry 176, 86–90. doi: 10.1192/BJP.176.1.86 10789334

[ref38] Zislin J. Katz G. Raskin S. Strauss Z. Teitelbaum A. Durst R. (2002). Male genital self-mutilation in the context of religious belief: the Jerusalem syndrome. Transcult. Psychiatry 39, 257–264. doi: 10.1177/136346150203900208

